# Effect of High-Dispersible Graphene on the Strength and Durability of Cement Mortars

**DOI:** 10.3390/ma14040915

**Published:** 2021-02-15

**Authors:** Xiaoqiang Qi, Sulei Zhang, Tengteng Wang, Siyao Guo, Rui Ren

**Affiliations:** 1 Department of Civil Engineering, Qingdao University of Technology, Qingdao 266033, China; dumingqing@qut.edu.cn (X.Q.); wangyantumu@qut.edu.cn (T.W.); 2 Key Laboratory for Urban Underground Engineering of Ministry of Education, Beijing Jiaotong University, Beijing 100044, China; 3 School of Highway, Chang’an University, Chang’an 710061, China; renrui@chd.edu.cn

**Keywords:** graphene oxide, high-dispersion graphene, cement mortars, pore structure, strength, durability

## Abstract

Graphene’s outstanding properties make it a potential material for reinforced cementitious composites. However, its shortcomings, such as easy agglomeration and poor dispersion, severely restrict its application in cementitious materials. In this paper, a highly dispersible graphene (TiO_2_-RGO) with better dispersibility compared with graphene oxide (GO) is obtained through improvement of the graphene preparation method. In this study, both GO and TiO_2_-RGO can improve the pore size distribution of cement mortars. According to the results of the mercury intrusion porosity (MIP) test, the porosity of cement mortar mixed with GO and TiO_2_-RGO was reduced by 26% and 40%, respectively, relative to ordinary cement mortar specimens. However, the TiO_2_-RGO cement mortars showed better pore size distribution and porosity than GO cement mortars. Comparative tests on the strength and durability of ordinary cement mortars, GO cement mortars, and TiO_2_-RGO cement mortars were conducted, and it was found that with the same amount of TiO_2_-RGO and GO, the TiO_2_-RGO cement mortars have nearly twice the strength of GO cement mortars. In addition, it has far higher durability, such as impermeability and chloride ion penetration resistance, than GO cement mortars. These results indicate that TiO_2_-RGO prepared by titanium dioxide (TiO_2_) intercalation can better improve the strength and durability performance of cement mortars compared to GO.

## 1. Introduction

The development of cement as the most basic material in civil engineering was more than 100 years, and today, it remains the most commonly used construction material [1,2]. With the increasing popularity of super high-rise, large span (sea-crossing bridges), and specially shaped buildings, the requirements for cement performance and durability are increasing. During the hardening stage for cement forming, cracks and voids are easily left in the structure [3]; these characteristics reduce the bearing capacity of the cement and accelerate the erosion effect of corrosive media, thus reducing the service life of the cement structure. Nowadays, the performance of cement has been improved by many efforts such as adjusting the cement mortars ratio, optimizing the aggregate gradation, and adding reinforcements [4,5,6,7]. However, these do not change the structure of cement hydration products, and the problems of high brittleness and cracking of the cement mortars still exist.

Nanomaterials have excellent physical and chemical properties and have been widely used in the field of cementitious composites [8,9,10,11]. Nanomaterials not only improve the macroscopic properties of cement mortars, but also regulate the crystalline morphology of cement hydration products through nucleation [12], thus achieving control of the hydration process of cement. Additionally, the nanomaterials are very small in size and dispersed in the cement mortars, which can fill the internal pores of the cement mortars [13] and improve the compaction of cementitious materials. Due to its excellent properties, graphene, a novel nanomaterial, shows important promise in many fields such as electronics, photonics, biophysics, and materials science [14,15,16,17,18], and it also has great potential for application in cement composites [19,20].

The introduction of graphene can effectively enhance the strength and durability of cement. It is found that the compressive strength of cement increases with increasing admixture content, and that smaller and thinner graphene oxide can significantly increase the compressive strength of cement [1,21,22,23,24]. Tong et al. [25] studied the effects of graphene and graphene oxide on the freeze–thaw resistance of cement mortars, and the test results show that there are different types of graphene that could enhance the freeze–thaw resistance of cement mortars. Mohammed et al. [26,27] found that adding a low percentage of graphene oxide (0.01%) could change the pore structure of the cement matrix, thus effectively preventing the ingress of chloride ions. Graphene can significantly improve the performance of cement mortars, but its poor dispersion seriously restricts the enhancement effect. The ultrasonic dispersion method can obtain a relatively homogeneous dispersion; however, it can damage the surface structure of graphene and affect its performance [28,29]. Graphene obtained by microwave exfoliation typically does not exceed 10 layers [30]. Non-covalent bonding modification can also be used to improve the dispersion of graphene by surface modification of graphene. Non-covalent bonding modification maintains the original structure and anisotropy of graphene as much as possible, which is more beneficial than covalent bonding modification for the application of graphene in various materials [8,31].

Based on the above problems, RGO was prepared using a modified Hummers method and ultrasonic treatment and converted into TiO_2_-RGO through the intercalation of TiO_2_. Tests such as UV-vis spectroscopy and Raman are performed to characterize and compare TiO_2_-RGO and GO. The prepared TiO_2_-RGO and GO are mixed into cement mortars in the same proportion, respectively. FT-IR and MIP tests were used to test the effect of GO or RGO-TiO_2_ on cement mortar hydration. The compressive and flexural tests are conducted separately to investigate the difference of the strength of TiO_2_-RGO- and GO-modified cement mortars. The effect of TiO_2_-RGO and GO in improving the durability of cement mortars is investigated through water absorption and chloride penetration tests. This study will help to promote the development of cementitious composites and nanomaterials, and significantly expand the scope of engineering applications of graphene/cement matrix composites.

## 2. Experiment

### 2.1. Materials

The Portland cement used in the experiment is P.O. 42.5, supplied by Shandong shanshui Cement Co. Ltd. (Qingdao, Shandong, China). Its chemical and physical parameters are shown in Table 1 and Table 2, respectively. The standard sand used in this study was supplied by China ISO Standard Sand Co., Ltd. (Xiamen, China), and the gradation of the standard sand is listed in Table 3. The yellow GO was synthesized via modified Hummer’s method [32] and ultrasonic treatment. The sol–gel method was used to prepare the titanium dioxide nanoparticle dispersant; then high-dispersion graphene (TiO_2_-RGO) was prepared by titanium dioxide intercalation during GO reduction process in the laboratory.

### 2.2. Preparation of Modified Cement Mortars

The mix proportions of cement mortar specimens are given in Table 4. Cement was added to the cement mixer, then distilled water was mixed with 0.01 wt%, 0.03 wt%, and 0.05 wt% GO or TiO_2_-RGO by weight of cement (marked as Mg-0.01, Mg-0.03, Mg-0.05, Mtg-0.01, Mtg-0.03, and Mtg-0.05, respectively), which were added to the cement and stirred for 1 min. The weighed standard sand was then poured into cement and the mixture was stirred for 1 min and 30 s at low and high speeds, respectively. The mixed cement was poured into prismatic molds and the mold was vibrated for 5 min to remove the entrapped air. The samples were covered with plastic wrap to avoid moisture escape. After 24 h, the cement mortar samples were removed from the mold and cured at 20 ± 2 °C and relative humidity RH ≥ 95% until strength test.

## 3. Test Methods

### 3.1. UV-Vis Spectroscopy and Raman Test

To further investigate the properties of TiO_2_-RGO, it was characterized by UV-vis spectroscopy (PerkinElmer, Boston, MA, USA) and Raman (Renishaw, London, UK). The TiO_2_-RGO solution and GO solution were fully stirred for 2 h with ultrasound and tested on a UV-vis spectrophotometer with a wavelength range of 200–800 nm. The TiO_2_-RGO solution was vacuumized in order to prepare the Raman test sample; after filtration two times, the sample was dried in air and a small portion of the filter paper was cut out for Raman testing, with a frequency range of 50–2500 cm^−1^.

### 3.2. MIP Test

Mercury intrusion porosimetry (MIP) (Quantachrome, Boynton Beach, FL, USA) was used to probe the pore structure parameters, and the test can be used to investigate the effect of TiO_2_-RGO and GO on the pore structure of cement mortars due to the wide range of measurements it produces (pore characteristics such as pore size distribution curves, porosity, and critical pore size). After standard curing for 28 days, the samples were demolded and broken into small pieces with a diameter of 5 to 10 mm and a weight of about 1 g using a tool. The samples were immersed in anhydrous ethanol to stop the cement mortars’ hydration and stored hermetically. The samples were dried to constant weight in an oven at 60 ± 2 °C and then cooled to room temperature in the drying oven to remove all foreign fluids from pores without affecting the pore structure. MIP (Auto Pore-master-33, Konta company, Boynton Beach, FL, USA) was used to examine the pore structure characteristics of the size range (5–360,000 nm) of the cement-based materials at 28 days. The principle is to calculate the diameter of a pore and the volume of different pores as a function of the volume of mercury intruding into the material and the applied pressure. The relationship between external pressure P and cement mortars pore radius r is as follows:

(1) $$r=\frac{-2\sigma \cos\theta }{p}$$

where *r* is the radius of the pore; *σ* is the surface tension of mercury; *θ* is the contact angle between the sample surface and mercury; *p* is the total external pressure on mercury.

The porosity of cement mortars is calculated as follows:

(2) $$\rho =\frac{V_{1}}{V_{2}}\times 100\%$$

where $V_{1}$ is the actual volume of mercury pressed into the sample and $V_{2}$ is the apparent volume of the sample.

### 3.3. FT-IR Test

The FT-IR scans were not only used to evaluate the functional groups of graphene and cement-based composite but also to monitor the hydration process of the cement mortars. FT-IR scans are generally used to test for functional groups of graphene, but they can also be used to determine changes in cement mortars’ hydration products. Therefore, in order to study the effect of TiO_2_-RGO and GO on cement mortars’ hydration products, in this paper, the composition of samples was investigated by Fourier transform infrared (FT-IR) spectroscopy using a spectrometer (Thermo Fisher Scientific, Waltham, MA, USA) over the wavenumber from 400 to 4000 cm^−1^.

### 3.4. Mechanical Strength Test

To examine the effect of incorporating the TiO_2_-RGO and GO mechanical properties of cement mortars, in this test, a group of samples of cement mortars without graphene was designed as a blank group. Six groups of cement mortar samples were designed with different admixture amounts of 0.01, 0.03, and 0.05 wt% for GO and TiO_2_-RGO, respectively. Cement mortar specimens were poured into the 40 × 40 × 160 mm mold for the flexural strength and compressive strength of cement mortars at the curing periods of 3, 7, and 28 days. This test required 7 sets of cement mortar samples: one set contained 3 mortar samples, and an average of the strengths of the 3 samples.

### 3.5. Water Absorption and Chloride Penetration Tests

**Figure 1:**
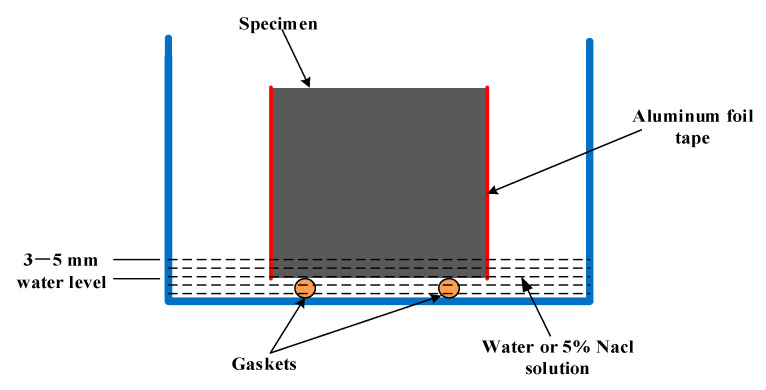
Schematic diagram of cement mortars immersion.

Following the standard test method proposed by Bamforth [33], water absorption and chloride penetration tests were performed in more detail in this paper. Cement mortar samples were demolded and dried after 28 days of standard curing, the aluminum foil tape was used to seal the samples, leaving only the top and bottom surfaces, and the gap between the tape and the underside was filled with paraffin. Then, samples were dried in an oven at 60 ℃ to constant weight to ensure that the water in the sample evaporated completely [34]. The mass of each cement mortar sample was weighed and the mass of each group of samples was averaged as the initial mass. Cement mortar samples were immersed in water and 5% NaCl solution for water absorption and chloride permeation tests, respectively. Gaskets were used at the bottom of the sample to maintain the water level of 3–5 mm above the top of the gaskets. A schematic diagram is shown in Figure 1.

At 0.5 h, 1 h, 2 h, 4 h, 8 h, 12 h, 24 h, 3 days, and 7 days intervals for the water adsorption test, the weight of the mortar samples was measured and the mass change was recorded at the same time. For the chloride permeation test, the chloride content of the specimens at different depths was determined by soaking the specimens in 5% NaCl solution for 28 days and then removing them. The cement mortar specimens were dried with the oven for internal moisture, sanded every 2 mm with a grinding machine, then sieved with a 0.63 mm fine sieve and sealed for storage, and the grinding depth of each mortar sample was 20 mm. Cement mortar samples were titrated by silver nitrate titration as specified in JTJ270-98; 2 g of sample powder was placed in 50 mL of distilled water and shaken for 30 min, after which it was left to stand for 24 h. The above 20 mL of liquid was filtered and put into a measuring cylinder; 2 drops of phenolphthalein solution were added and the color of the solution changed to rosy red. The solution was again neutralized by the dilute sulfuric acid added and became colorless, then 10 drops of potassium chromate solution were dropped into the solution. Finally, silver nitrate was dropped into the above solution and the volume used to turn the solution brick red was recorded. The formula for calculating the content of chloride ions was as follows:

(3) $$P=\frac{C\times V_{5}\times 0.03545}{G\times \frac{V_{4}}{V_{3}}}\times 100\%$$

where P is the chloride ion content in powder (%); C is the standard concentration of AgNO_3_ solution (mol/L); G is the mass of the cement mortar sample powder (g); $V_{3}$ is the amount of water used to dissolve the specimen powder (mL); $V_{4}$ is the amount of filtrate required for titration (mL); $V_{5}$ is the amount of silver nitrate solution for titration (mL).

## 4. Results and Discussion

### 4.1. UV-Vis Spectrum of GO and TiO_2_-RGO

**Figure 2:**
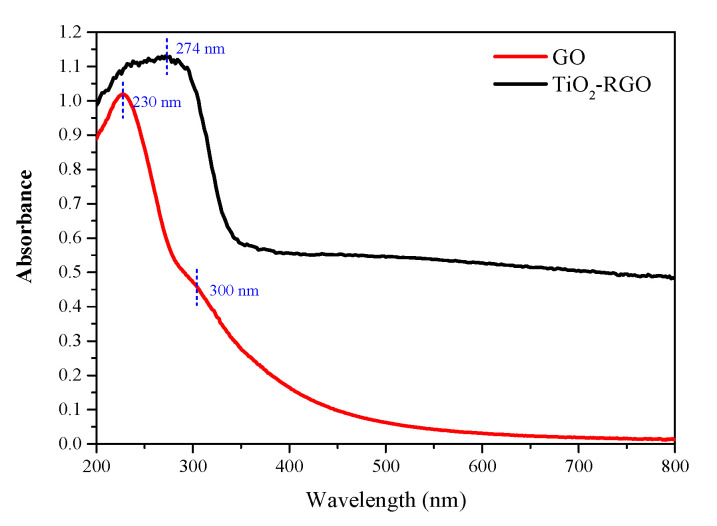
UV-vis spectra of GO and TiO_2_-RGO in water.

The dispersion of graphene can be evaluated by UV-vis spectroscopy [35]. Figure 2 shows UV-vis spectra of GO and TiO_2_-RGO in water. The results show that there are two peaks in the UV-vis spectra curve of GO, where the peak at 228 nm is the π–π* electron jump of the C=C bond and the peak at 300 nm is the n–π* electron jump of the C=O double bond. It is the same location of the UV-vis characteristic absorption peak that has been reported for GO [36]. These two peaks reflect the dispersion and reduction of GO. It can be found that there is only one peak in the UV-vis spectra of TiO_2_-GO and it is shifted, where the absorption peak red shifts to 274 nm, while the 300 nm absorption peak of GO disappears, compared to the GO spectrum. The redshift of the absorption peak at 230 nm is the result of recombination of the π–π covalent bond, while the disappearance of the absorption peak at 300 nm is the result of the reduction of the C=O double bond, which shows that GO has been reduced to TiO_2_-RGO.

The absorbance is proportional to the number of particles per unit volume according to the equation, as shown in Equation (4). The higher absorbance means a higher concentration of dispersion and more stable dispersion. The UV-visible spectrum peak of TiO_2_-RGO is higher than that of GO, which means that TiO_2_-RGO has greater absorbance and better dispersion compared with GO.

(4) A=K×V

where *A* is the absorbance; *K* is the absorbance constant, which is related to the nature of the absorbed material and the incident wavelength; *V* is the number of particles per unit volume.

### 4.2. Raman Analysis

**Figure 3:**
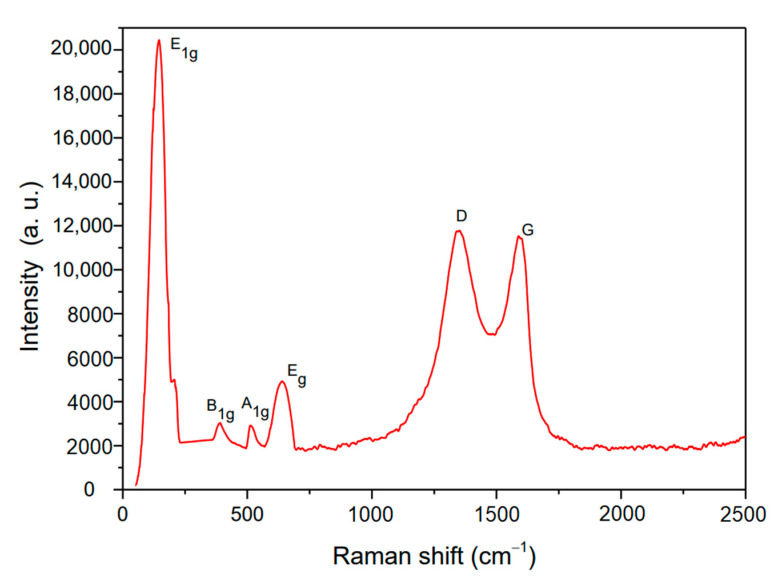
Raman spectroscopy of the TiO_2_-RGO.

Graphene component and structural defects are often judged by Raman spectroscopy. To further characterize the molecular structure of the TiO_2_-RGO used in this study, identification of the TiO_2_-RGO was performed with Raman spectroscopy, as shown in Figure 3. It can be seen from the figure that there are E1g, B1g, A1g, and Eg absorption peaks of anatase phase TiO_2_ in 146, 392, 512, and 638 cm^−1^, indicating that anatase TiO_2_ is contained in the TiO_2_-RGO solution [37]. The Raman spectrum indicates the D-band at 1341 cm^−1^ and the G-band at 1599 cm^−1^, which are the main characteristic peaks of graphene. The D-band represents the disordered vibration peak of the TiO_2_-RGO, and the G-peak is the vibration peak of the SP^2^ carbon atom surface. The ratio of the strength of the two characteristic peaks D and G is small, indicating that TiO_2_-RGO has excellent reducibility and fewer crystal defects. This result is the same as the UV-vis spectrum of TiO_2_-RGO, which indicates that TiO_2_-RGO has been completely reduced.

### 4.3. Pore Structure

Cement mortar is a porous material compounded by multiple components, and its strength and permeability are closely related to the internal microstructure such as the pore size distribution of the system. Therefore, the pore structure of cementitious materials is an important parameter to study their performance. The mercury intrusion porosity test is carried out for the blank group cement mortars and the modified cement mortars mixed with 0.03 wt% TiO_2_-RGO or GO, respectively. There are many definitions of cement-based materials at home and abroad, and these are most commonly divided into four categories: gel pores, capillary pores, macropores due to deliberately entrained air, and macropores due to inadequate compaction [38]. The pore system in the capillary pore consists of three types of pores: 2.5–10, 10–50, 50–10,000 nm, which correspond to small isolated capillaries, medium capillaries, and large capillaries, respectively [39].

**Figure 4:**
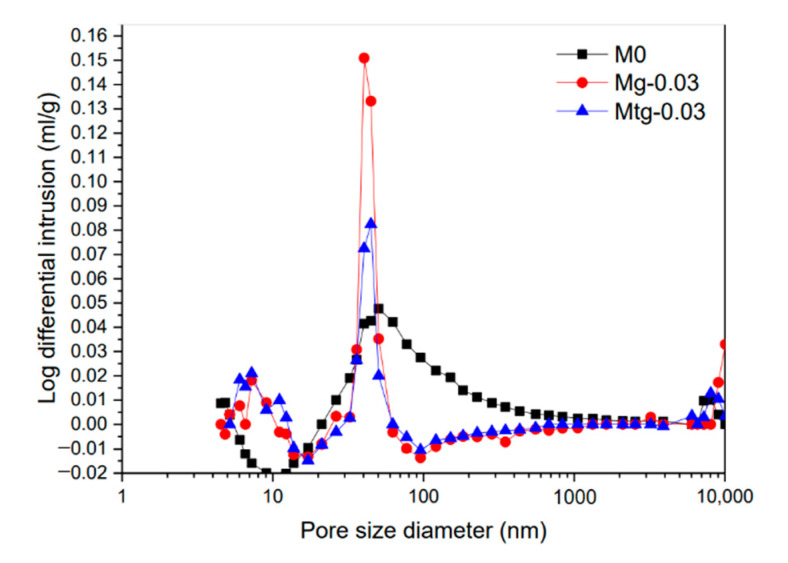
The pore size distribution of different cement mortar mixtures.

Figure 4 shows the pore size distribution of cement mortar samples with GO content of 0.03 wt%, TiO_2_-RGO content of 0.03 wt%, and blank cement mortar samples. It can be seen that cement mortars mixed with TiO_2_-RGO or GO had larger contributions at small isolated capillaries and both are larger than the blank group, while the contributions at large capillaries are opposite to the above results.

**Figure 5:**
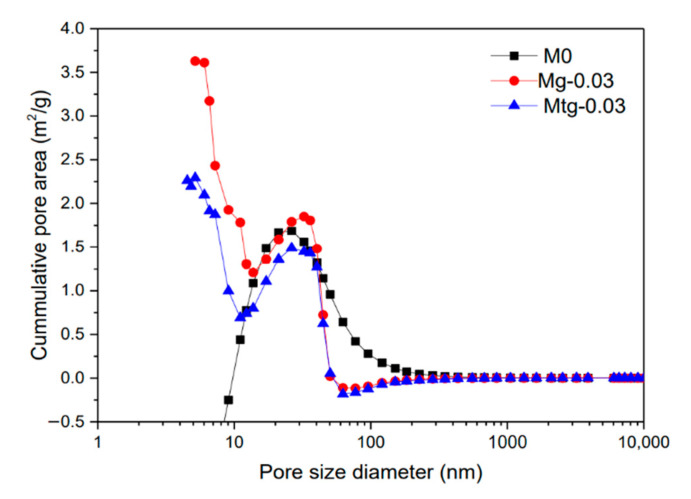
Cumulative pore area of different cement mortar mixtures.

The trend of cumulative surface area of different cement mortar samples is shown in Figure 5. The cumulative pore surface area of the TiO_2_-RGO cement mortars and GO cement mortar samples is smaller than that of the blank group when the pore size is less than 10 nm, while the opposite is true when the pore size is greater than 50 nm. This indicates that the pore size of cement mortars with TiO_2_-RGO and GO is generally smaller than that of the blank test group; the addition of TiO_2_-RGO or GO perfects the pore structure of cement mortars and improves compactness. In the figure, the peak value of the curve belongs to the most probable pore size, featuring the highest probability of pore size in the cement mortars. The probable pore size of GO-doped cement mortars is 50 nm with a mercury input of about 0.155 mL/g, and the probable pore diameter of cement mortars with TiO_2_-RGO is 40 nm with a mercury input of about 0.09 mL/g. The cumulative void’s specific surface area of cement mortal samples doped with TiO_2_-RGO is smaller than that of cement mortar samples doped with GO, and the difference is larger for pore sizes smaller than 20 nm. Moreover, compared with the blank group, the cement mortars with TiO_2_-RGO have a larger reduction in porosity. It is shown that TiO_2_-RGO can refine the pore structure better and reduce the pore size of cement mortars compared with GO.

Table 5 shows the parameters of the mercury intrusion analysis with GO content of 0.03 wt%, TiO_2_-RGO content of 0.03 wt%, and blank cement mortar samples. With the MIP test, it can be seen that the average diameters of cement mortars with GO and TiO_2_-RGO are much lower than those of the blank group, and cement mortars with TiO_2_-RGO are the smallest. The porosity of cement mortars with GO and TiO_2_-RGO is measured to be 4.3%, 3.2%, and 2.6% for blank groups, respectively. Obviously, compared with the blank group, the porosity of cement mortars containing GO and TiO_2_-RGO decreased by about 26% and 40%, respectively. This is consistent with the results of previous studies [40,41]. From the average pore size and porosity, it can be seen that the effect of adding TiO_2_-RGO is significantly better than GO.

### 4.4. FT-IR Analysis

**Figure 6:**
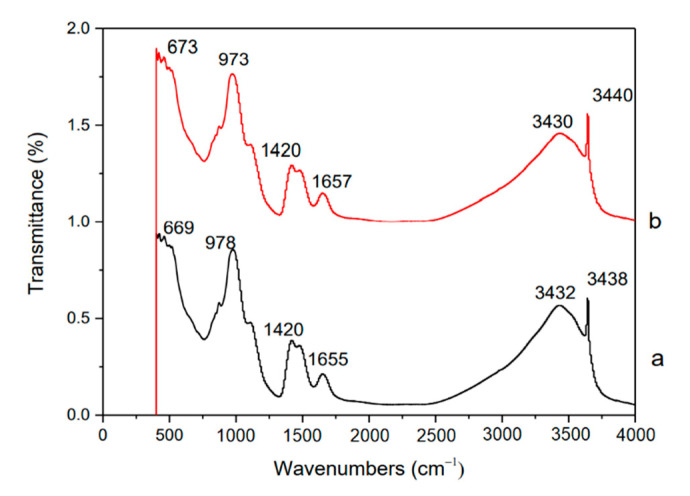
FT-IR spectrum of modified cement mortars. a—blank cement mortar sample. b—0.03wt% TiO_2_-RGO cement mortars sample.

In order to further investigate the effect of TiO_2_-RGO on cement mortars’ hydration process, the hydration products of modified cement mortars are analyzed by Fourier-transform infrared spectroscopy. Figure 6 represents the FT-IR spectrum of hydration products of blank sample at 28 days. The peak at 3438 cm^−1^ is the absorption peak (-OH) in the hydration product Ca(OH)_2_, the absorption peak for the calcium sulfate and calcium carbonate are observed at 3432 and 1655 cm^−1^ wavelengths, and the absorption peak at 669 cm^1^ represents the hydration product α-C_2_S [42]. Figure 6 represents the FT-IR spectrum of hydration products of the modified cement mortars with TiO_2_-RGO content of 0.03% at 28 days; absorption peaks are found at 3640, 3430, 1657, 1420, 972, and 673 cm^−1^, respectively. The FT-IR spectrum of the cement mortar samples modified with TiO_2_-RGO is similar to those of the blank samples, and it has no new absorption peaks. This shows that TiO_2_-RGO only plays a physical regulatory role and does not participate in the chemical reaction to produce a new type of hydration product when TiO_2_-RGO is added to cement mortars, though it can participate in the hydration process of cement mortars.

### 4.5. Compressive and Flexural Strength of Modified Cement Mortars

Many pores exist in the hydrated cement mortars, and a large number of pores in the cement mortars make the substrate poorly compacted and reduce the bearing capacity, which is one of the reasons for the low cement mortars strength. It has been reported in the literature that the use of graphene-modified cement mortars can improve its mechanical properties, and TiO_2_-RGO prepared by titanium dioxide intercalation has better dispersion properties than GO. Therefore, to investigate the effect of GO and TiO_2_-RGO on the strength of cement mortars, the compressive and flexural strengths of GO cement mortars and TiO_2_-RGO cement mortars are tested separately.

**Figure 7:**
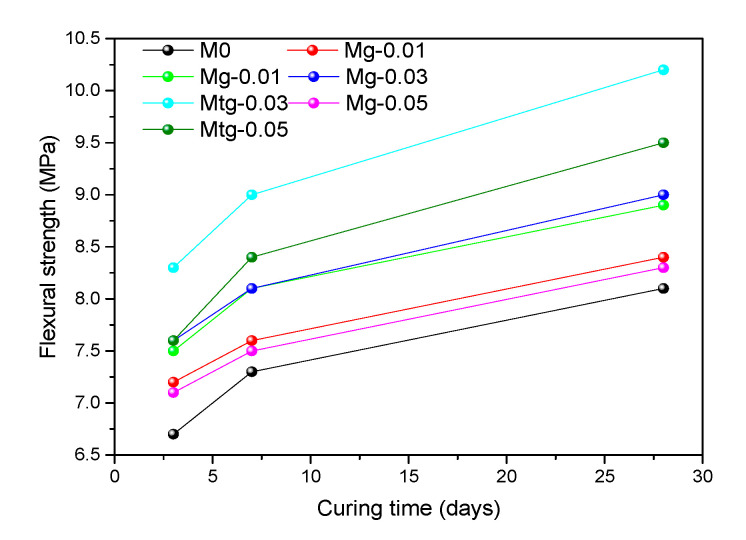
Compressive strength of modified cement mortars.

**Figure 8:**
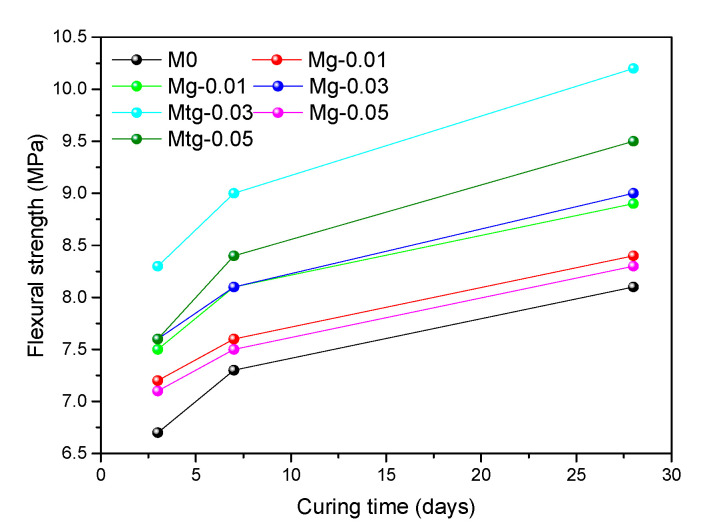
Flexural strength of modified cement mortars.

Figure 7 and Figure 8 present the compressive and flexural strengths of untreated and modified cement mortars over time. The results show that the compressive strength of the samples added with GO or TiO_2_-RGO is greater than that of the blank samples. Compared with the blank samples, the 28-day compressive strengths are improved by about 6.1%, 11.7%, 8.9%, 8.5%, 28.0%, and 17.4% for the groups Mg-0.01, Mg-0.03, Mg-0.05, Mtg-0.01, Mtg-0.03, and Mtg-0.05, respectively, and the 28-day flexural strengths with the addition of GO and TiO_2_-RGO are improved by 2% to 11% and 9% to 24%, respectively. Wang [43] and Lv [44] reported that GO can modulate hydration to form dense flower-like structures, thereby enhancing the strength of cement mortars. This could be linked to the aforementioned discussion about the MIP test results where GO or RGO-TiO_2_ cement mortars are denser, less porous, and therefore stronger. GO and TiO_2_-RGO both regulate cement mortar hydration by providing templates and growing nuclei for cement mortar hydration, which is beneficial to develop dense structures, thereby greatly increasing the compressive and flexural strength of cement mortars.

In addition, the strength of cement mortars changes considerably after mixing with GO and TiO_2_-RGO, with its compressive strength and flexural strength increasing almost twice as much as that of the samples modified with GO. The primary contributor to this difference may be that TiO_2_-RGO has better dispersion properties than GO, as demonstrated by the UV-vis spectroscopy test. TiO_2_-RGO is more uniformly and stably dispersed in the cement mortars, thus allowing TiO_2_-RGO to better regulate the hydration of cement mortars. In addition, the TiO_2_-RGO mixed with nano-TiO_2_, which itself has a high strength, thus greatly enhancing the strength of cement mortars.

Furthermore, as shown in Figure 7 and Figure 8, the compressive and flexural strengths increased with increasing TiO_2_-RGO concentration but only up to 0.03%; further increases in the TiO_2_-RGO concentration weakened this reinforcing effect and reduced the strength of the cement mortar.

Through comparative testing, 0.03 wt% was found to be the optimal concentration of TiO_2_-RGO in cement mortars. This is mainly due to the large amount of TiO_2_-RGO admixture that will result in easy caking after mixing with cement mortars, which seriously affects the effect of TiO_2_-RGO on the strength of cement mortars. These phenomena are also found in the compressive and flexural strength of the GO cement mortar samples and are caused by the same reasons as above.

### 4.6. Water Absorption

**Figure 9:**
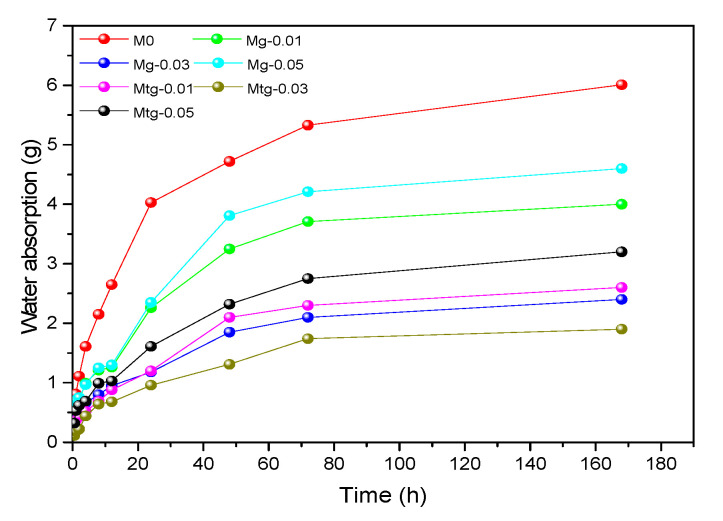
Accumulated water absorption curve of modified cement mortars.

The durability of cement mortars is closely related to its water absorption rate, and the failure of cement mortars mostly occurs in the presence of water and harmful substances; therefore, the water absorption test is an important test method to evaluate and reveal the durability of cement materials. Figure 9 shows the curves of water absorption rate of cement mortars with different amounts of GO and TiO_2_-RGO with time. The results show that with the increase in capillary water absorption time, the capillary water absorption of all samples accumulates, but the capillary water absorbability gradually decreases, and the water absorbability of cement mortars mixed with GO or TiO_2_-RGO is much lower than that of blank samples. The capillary water absorbability tended to decrease with increasing GO content, with the lowest water absorbability occurring at 0.03 wt%; the same phenomenon occurs in the cement mortar samples mixed with TiO_2_-RGO. In addition, the water absorption rate of the TiO_2_-RGO-doped cement mortar samples was lower than that of the GO-doped cement mortar samples at the same GO and TiO_2_-RGO contents.

The coefficient of capillary water absorption is often used as an indicator of water absorption. Experiments on capillary water absorption conducted in a short period of time show that, without considering the effect of gravity on water, there is an approximate linear relationship between the water absorption rate per unit area of the sample and the square root of the water absorption time [45], the formula of which can be expressed as follows:

(5) $$\Delta W=A\sqrt{t}$$

where ΔW is water absorption per unit area of sample (g/m^2^); *A* is the coefficient of capillary water absorption of the sample (g/m^2^·h^1/2^); t is the water absorption time of sample (h).

**Figure 10:**
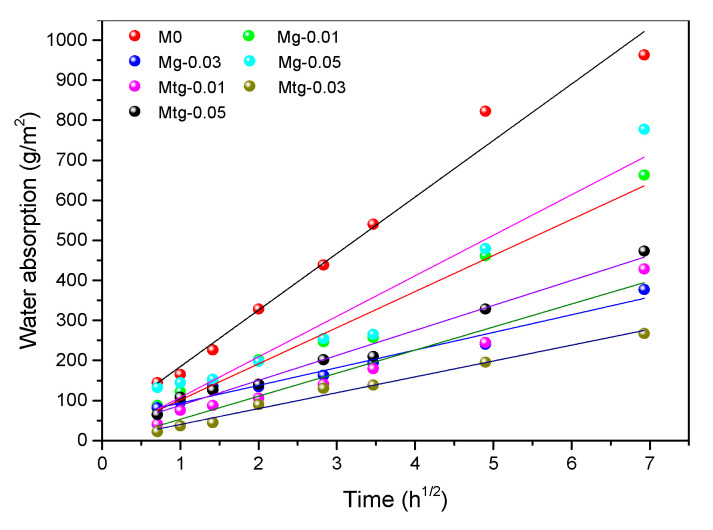
Fitting curve of capillary water absorption coefficient of modified cement mortars.

The linear fitting of capillary water absorption curves for the samples over 48 h and the fit curve of the capillary water absorption coefficient is shown in Figure 10; specific capillary water absorption coefficients are shown in Table 6. It can be found that when the GO or TiO_2_-RGO content is 0.03 wt%, the capillary water absorption coefficient of the samples is the smallest, which is 44.088 and 39.591, respectively. The samples had 68.77% and 71.96% lower capillary water absorption coefficients, respectively, compared with the blank samples. In addition, TiO_2_-RGO cement mortar samples showed lower capillary water absorption than the GO cement mortar samples with the same content.

The main reason for this phenomenon is that the pores of GO- or RGO-TiO_2_-doped specimens are smaller and the pore size distribution is more uniform than that of blank specimens. In addition, the MIP test results show that the sample doped with RGO-TiO_2_ has a smaller porosity than the sample doped with GO, which also explains its stronger water permeability resistance. It has been shown that graphene can optimize the pore structure distribution of cement mortar, thus improving the resistance of cement mortar to water penetration [41,46].

### 4.7. Chloride Penetration

**Figure 11:**
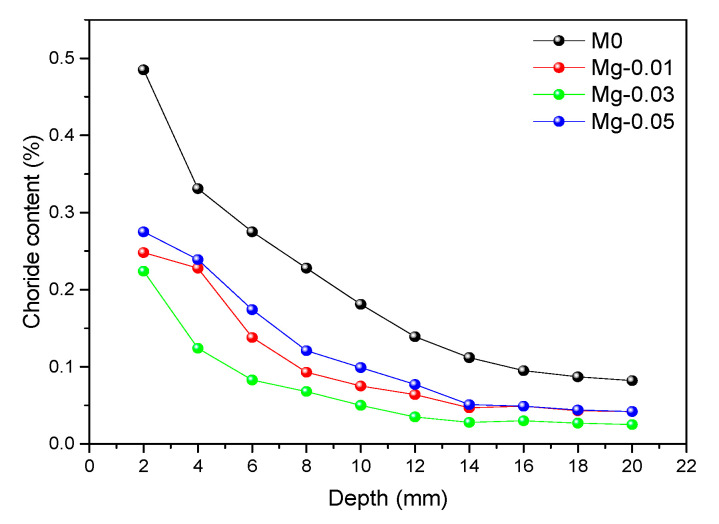
Chloride ion content of cement mortars mixed with GO.

**Figure 12:**
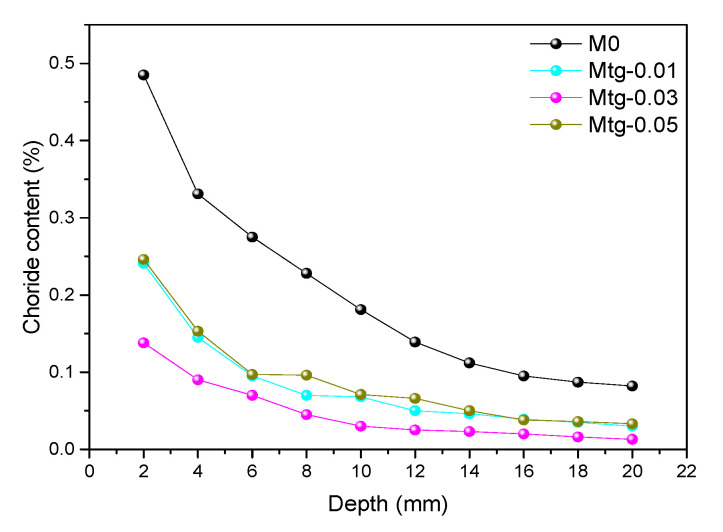
Chloride ion content of cement mortars mixed with TiO_2_-RGO.

Figure 11 shows the distribution curve of chloride ion content of GO cement mortar samples with different content. It can be seen that the chloride ion content of all cement mortar samples decreases with increasing depth. Notably, at the same depth of the sample, the chloride ion content of cement mortar samples decreased with increasing GO of up to 3%, and after it is increased, the chloride content in the graphene oxide sample is lowest at 0.03 wt% GO content and highest at 0.05% content, but is still lower than that of the blank sample at this depth. The distribution curves of chloride ion content of TiO_2_-RGO cement mortar samples with different content are shown in Figure 12. The distribution of chloride ion content of cement mortar samples with TiO_2_-RGO is the same as those mortar samples with GO, the chlorine ion content in the sample decreases with increasing depth of penetration, and the lowest internal chlorine ion content of the sample is found at 0.03 wt% TiO_2_-RGO content. Therefore, the results of this study clarify that adding GO/TiO_2_-RGO to cement mortar demonstrably improves chloride ion penetration resistance. This is in agreement with previous studies [26,47].

**Figure 13:**
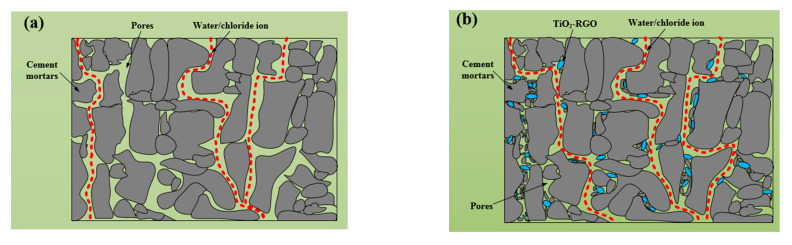
Cement mortars porosity diagram: (**a**) blank cement mortars; (**b**) TiO_2_-RGO cement mortars.

The reasons for this phenomenon can be seen in Figure 13. It is shown in Figure 13a that the existence of pores in cement mortars provides channels for the penetration of water and chloride ions. After adding TiO_2_-RGO to the cement mortars, the main reason for the increase in penetration resistance of the cement mortars is shown in Figure 13b. The size of TiO_2_-RGO is at nanoscale, which can fill the pores of the cement mortars. As shown in the figure, since water and chloride ions cannot pass through the pores of cement mortars filled with TiO_2_-RGO (as shown in Figure 13b), water in Figure 13a has changed its permeation pathways, resulting in longer permeation times [37,38]. Like graphene oxide, TiO_2_-RGO can provide adsorption sites for cement mortars’ hydration and accelerate the hydration rate of cement mortars [39]; in addition, the TiO_2_-RGO, as a template, can adjust the microstructure of hydration products to form a tight cross-linked structure [40], and it becomes more difficult for water to pass through the pores with the compactness of the cement mortars’ structure, as shown in Figure 13b. As chloride ion penetration takes water as the medium, and it intrudes into the cement mortar samples’ interior containing water through the action of capillary, the degree of chloride ion erosion is determined by the rate and amount of moisture movement within the mortar sample. The degree of chloride ion intrusion into the modified sample decreases due to GO/TiO_2_-RGO content which reduces the capillary water absorption of the mortar sample. The effect of different content of TiO_2_-RGO on the resistance of mortar samples to chlorine ion erosion is the same as the effect on capillary water absorption. At a content of 0.03 wt% of GO or TiO_2_-RGO, the mortar sample has the smallest capillary water absorbability, resulting in the smallest amount of chloride ions moving with water into the sample. In summary, the significant improvement in impermeability and chloride ion penetration resistance of the cement mortars was mainly due to the contribution of GO/TiO_2_-RGO, which optimized the distribution of the pore structure, reduced the porosity, and improved the compactness of the cement mortars.

**Figure 14:**
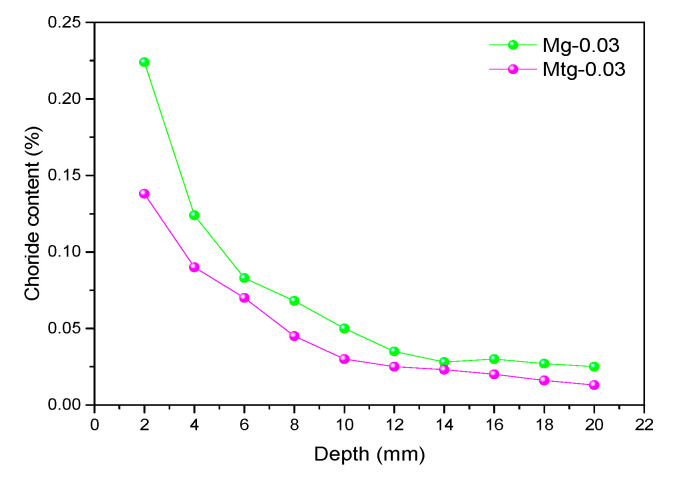
Chloride ion content of 0.03 wt% GO and TiO_2_-RGO.

In the above tests, the optimum content of GO and TiO_2_-RGO against chloride ion penetration for cement mortar samples was 0.03 wt%. Figure 14 shows a comparison of the chlorine ion content distribution of 0.03 wt% cement mortars with GO or TiO_2_-RGO. Obviously, it can be seen that the internal chloride ion content of the cement mortar samples doped with TiO_2_-RGO is lower than that of the cement mortar samples doped with GO at the same depth. The result indicates that TiO_2_-RGO can significantly improve the chloride ion resistance of cement mortars compared to GO. The main reason is that TiO_2_-RGO has a higher dispersion than GO, which can better regulate the hydration of cement mortars to form a dense structure and improve the water penetration resistance of cement mortar samples [48], thus further preventing chlorine ions from penetrating into the cement mortars, and enhancing the chloride ion corrosion resistance of cement mortar samples.

## 5. Conclusions

In this paper, GO and TiO_2_-RGO are prepared and characterized. Different concentrations of GO or TiO_2_-RGO were added to cement mortar, and the characteristics of the GO or TiO_2_-RGO and the mechanical and impermeability properties of the cement mortars were examined and compared with those of untreated cement mortars. The following conclusions are drawn:

1. The UV-vis spectroscopy test with GO and TiO_2_-RGO shows that TiO_2_-RGO can be uniformly dispersed in water and TiO_2_-RGO has better dispersion compared with GO. The Raman result demonstrates the successful incorporation of TiO_2_ between graphene sheets, which is the reason for the superior dispersion of TiO_2_-RGO.
2. The FT-IR results of cement mortars’ hydration products show that the content of GO and TiO_2_-RGO does not generate new products. The MIP test of modified cement shows that GO and TiO_2_-RGO can improve the distribution of pore structure in cement mortars, increase the number of small isolated capillaries and medium capillaries in cement mortars, reduce large capillaries, and decrease the porosity of cement mortars, thus improving the strength and durability of modified cement mortars. In addition, compared with GO, TiO_2_-RGO has superior effect in improving the pore structure.
3. The strength and durability tests of modified cement mortars show that the flexural and compressive strength, impermeability, and corrosion resistance of the modified cement mortars have been significantly improved. TiO_2_-RGO cement mortars have better performance than GO cement mortars at the same admixture amount. The optimal weights of GO and TiO_2_-RGO are both 0.03 wt% within the investigated range.

## Figures and Tables

**Table 1:** Physical properties of the Portland cement.

Compressive Strength	The Initial Setting Time	The Final Setting Time	Standard Consistence
42.5 MPa	2.33 h	4.01 h	28.6%

**Table 2:** Chemical composition of the cement.

Type	Content
Calcium oxide	64.5%
Silicon dioxide	19.1%
Aluminum oxide	4.95%
Sulfur trioxide	4.55%
Ferric oxide	3.53%
Magnesium oxide	1.83%
Loss on ignition	1.75%

**Table 3:** The size distribution of standard sand.

**Square Mesh Size (µm)**	80	160	500	1000	1600	2000
**Remaining on the Sieve (%)**	99 ± 1	87 ± 4	67 ± 4	33 ± 4	7 ± 4	0

**Table 4:** Cement mortars mixture proportions.

Num	Cement (g)	Standard Sand (g)	W/C	GO	TiO_2_-RGO
M0	450	1350	0.42	0	0
Mg-0.01	450	1350	0.42	0.01%	0
Mg-0.03	450	1350	0.42	0.03%	0
Mg-0.05	450	1350	0.42	0.05%	0
Mtg-0.01	450	1350	0.42	0	0.01%
Mtg-0.03	450	1350	0.42	0	0.03%
Mtg-0.05	450	1350	0.42	0	0.05%

**Table 5:** MIP analysis of different samples.

Group	Maximum Amount of Mercury (mL/g)	Average Diameters (nm)	Porosity (%)	Porosity Reduction (%)
M0	0.027	125	4.3	0
Mg-0.03	0.023	23	3.2	26
Mtg-0.03	0.019	17	2.6	40

**Table 6:** Capillary water absorption coefficient of modified cement mortars.

Sample	Regression < 48 h	R	Capillary Water Absorption Coefficient (g/m^2^ h^1/2^)
M0	y = 141.181 ×+ 7.833	0.979	141.181
Mg-0.01	y = 90.167 ×+ 5.768	0.972	90.167
Mg-0.03	y = 44.088 ×+ 3.101	0.966	44.088
Mg-0.05	y = 101.248 ×+ 10.141	0.934	101.248
Mtg-0.01	y = 57.582 ×+ 4.234	0.963	57.582
Mtg-0.03	y = 39.591 ×+ 1.871	0.985	39.591
Mtg-0.05	y = 62.417 ×+ 3.166	0.982	62.417

## Data Availability

All data has been included in the paper.
